# The First Isolation and Whole Genome Sequencing of Murray Valley Encephalitis Virus from Cerebrospinal Fluid of a Patient with Encephalitis

**DOI:** 10.3390/v10060319

**Published:** 2018-06-11

**Authors:** Jessica S. Russell, Leon Caly, Renata Kostecki, Sarah L. McGuinness, Glen Carter, Dieter Bulach, Torsten Seemann, Tim P. Stinear, Rob Baird, Mike Catton, Julian Druce

**Affiliations:** 1 Victorian Infectious Diseases Reference Laboratory at the Peter Doherty Institute for Infection and Immunity, 792 Elizabeth Street, Melbourne, VIC 3000, Australia; jessica.russell@mh.org.au (J.S.R.); leon.caly@mh.org.au (L.C.); renata.kostecki@mh.org.au (R.K.); mike.catton@mh.org.au (M.C.); 2 Department of Infectious Diseases, Royal Darwin Hospital, 105 Rocklands Drive, Tiwi, Darwin, NT 0810, Australia; sarah.mcguinness@monash.edu; 3 Doherty Applied Microbial Genomics, Department of Microbiology and Immunology, University of Melbourne, Doherty Institute for Infection and Immunity, 792 Elizabeth Street, Melbourne, VIC 3000, Australia; glen.carter@unimelb.edu.au (G.C.); dbulach@gmail.com (D.B.); t.seemann@unimelb.edu.au (T.S.); tstinear@unimelb.edu.au (T.P.S.); 4 The Microbiological Diagnostic Unit Public Health Laboratory, Department of Microbiology and Immunology, University of Melbourne, Doherty Institute for Infection and Immunity, 792 Elizabeth Street, Melbourne, VIC 3000, Australia; 5 Territory Pathology, Royal Darwin Hospital, 105 Rocklands Drive, Tiwi, Darwin, NT 0810, Australia; Rob.Baird@nt.gov.au

**Keywords:** Murray Valley encephalitis, *Flavivirus*, cerebrospinal fluid, isolation, whole genome sequencing

## Abstract

Murray Valley Encephalitis virus (MVEV) is a mosquito-borne Flavivirus. Clinical presentation is rare but severe, with a case fatality rate of 15–30%. Here we report a case of MVEV from the cerebrospinal fluid (CSF) of a patient in the Northern Territory in Australia. Initial diagnosis was performed using both MVEV-specific real-time, and Pan-*Flavivirus* conventional, Polymerase Chain Reaction (PCR), with confirmation by Sanger sequencing. Subsequent isolation, the first from CSF, was conducted in Vero cells and the observed cytopathic effect was confirmed by increasing viral titre in the real-time PCR. Isolation allowed for full genome sequencing using the Scriptseq V2 RNASeq library preparation kit. A consensus genome for VIDRL-MVE was generated and phylogenetic analysis identified it as Genotype 2. This is the first reported isolation, and full genome sequencing of MVEV from CSF. It is also the first time Genotype 2 has been identified in humans. As such, this case has significant implications for public health surveillance, epidemiology, and the understanding of MVEV evolution.

## 1. Introduction

Murray Valley Encephalitis Virus (MVEV) is a mosquito-borne member of the Flaviviridae family belonging to the Japanese Encephalitis serocomplex. It is an arbovirus of considerable public health importance in Australia due to the significant morbidity and mortality it may cause.

MVEV is enzootic to Northern Australia, existing in a complex transmission cycle in mosquitoes (*Culex* spp.) and water birds [1]; with periodic ‘dead-end’ involvement of other vertebrate hosts, including humans, where infection results in insufficient replication to perpetuate the viral life cycle [2]. Consequently, the incidence of MVEV outside of Northern Australia follows the migration of infected carrier birds, and is influenced by meteorological factors such as heavy rainfall, temperature, and humidity, which facilitate widespread movement of waterbirds and increase mosquito population [3]. This was exemplified by an outbreak in 2011 where seasons of excessive rainfall and flooding widened the migration grounds of waterbirds and allowed for the passage of MVEV into South-Eastern Australia [4,5].

Infection with MVEV is generally asymptomatic, or characterized by a mild, non-specific febrile illness, with only 1/150–1/2000 infections resulting in clinically apparent disease [3,4,6]. However, when it does occur, disease is frequently severe with a case fatality rate of 15–30% and long-term neurological sequelae persisting in 30–50% of survivors [4,6]. Several epidemics have been recorded in Australia in the past century, most notably in 1974 where 58 cases and 4 deaths were reported. Since then, a further 127 human cases have been recorded [5], including 17 cases in a 2011 outbreak where only 4 out of 17 patients achieved a full recovery [4].

As no vaccine or treatment exists for MVEV, outcomes are reliant on early diagnosis and good supportive care in Intensive Care Units [6]. Diagnosis of MVEV often relies heavily on serology, the interpretation of which may be challenging due to cross-reactivity of anti-Flavivirus antibodies [6]. Furthermore, in the absence of IgM, it is important to demonstrate increasing levels of IgG [3]. Radiological imaging may provide useful supporting information which is faster than serology [6] but not specific for MVEV.

At present, virological detection of MVEV is generally performed by PCR, which is favored due to its speed and sensitivity compared to traditional methods of virus isolation. Within our laboratory, detection of flaviviruses is performed using a nested pan-*Flavivirus* PCR targeting the whole Flaviviridae family, plus a species-specific real-time PCR, where available. Rapid identification of MVEV is achieved using an MVEV-specific real-time PCR, and verified by sequencing of the pan-*Flavivirus* PCR product.

Traditionally, MVEV has proved difficult to isolate from clinical specimens. All recorded isolations have been derived from post mortem brain tissue, and only one successful isolation has been reported since 1974 [3,7,8].

To date, only five MVEV isolates, four of them isolated over 40 years ago, have been sequenced using next generation sequencing technologies [1,8]. Accordingly, there is limited genomic information relating to active strains currently circulating in Australia. Four genotypes of the virus are currently recognized. In Australia type 1 has been identified as the dominant and most virulent genotype, with type 2 considered less virulent, and types 3 & 4 are extinct/non-circulating [5].

In this study, we describe detection by PCR, isolation, and full genome sequencing of a contemporary MVEV from a living patient’s cerebrospinal fluid (CSF), with identification of the genotype as type 2. This is the first reported isolation of MVEV from CSF and has significant implications for public health surveillance, epidemiology, and understanding of MVEV evolution.

## 2. Materials and Methods

### 2.1. Patient/Clinical Details

An 8-year-old previously healthy child, with no prior medical manifestations, was transferred from a remote Northern Territory community to the Royal Darwin Hospital with fever, seizures, and altered consciousness. Following direct admission to the intensive care unit, intubation was performed to facilitate lumbar puncture and magnetic resonance imaging (MRI). CSF analysis revealed an elevated CSF protein of 0.86 g/L (NR 0.15–0.45 g/L), CSF glucose of 2.7 mmol/L (NR 2.7–4.2 mmol/L) and a leucocytosis (388 × 10^6^/L) with a polymorphonucleocyte predominance (80%). Gram stain, India ink, and bacterial culture were negative. MRI of the brain revealed diffuse leptomeningeal enhancement with T2 hyperintensity and restricted diffusion involving the left basal ganglia and bilateral thalami, a pattern of involvement suspicious for flavivirus encephalitis. Electroencephalography demonstrated diffuse slowing without focal epileptiform features, consistent with infective encephalitis. Following extubation, observed neurological deficits included spastic paraplegia, dystonia and deficits in self-care, language, and cognitive domains. The patient required a prolonged hospital admission and 12 weeks of inpatient rehabilitation, but experienced a near-complete neurological recovery and was able to return home.

### 2.2. Nucleic Acid Extraction, Reverse Transcription, PCR Detection, and Sequencing

CSF from the patient was received in our laboratory in May 2015 from the Royal Darwin Hospital, accompanied by a request for MVEV PCR. Pan-*Flavivirus* conventional PCR and MVE-specific real-time PCR were performed according to our standard laboratory algorithm for diagnosis. Prior to extraction the specimen was treated with lysis buffer spiked with low copy number bovine diarrheal virus (BVDV) to act as internal control for the nucleic acid extraction, reverse transcription, and PCR amplification. This approach of using a non-human virus to act as an internal control has been previously described by our laboratory [9,10]. Viral nucleic acid was extracted from 200 µL patient CSF using the Qiagen QiaCUBE automated extraction robot with Qiagen reagents and consumables (Qiagen, Hilden, Germany) and eluted in 70 µL AVE elution buffer as per the manufacturer’s instructions. 10 µL of nucleic acid extract underwent reverse transcription using the SensiFast cDNA synthesis kit (BIOLINE, London, UK) as per the manufacturer’s instructions.

The conventional PCR utilizes highly degenerate pan-*Flavivirus* primers, modified from Scaramozzino et al., 2001 which target the NS5 region [11]. 2.5 µL of cDNA template was added to the master mix containing 0.5 µM of first or second round primer pool, 1.8 mM Mg Cl_2_, 2 µM dNTPs and 0.3 U of Taq polymerase (Qiagen) to a final volume of 40 µL. First round PCR cycling conditions consisted of one cycle of 94 °C for 3 min, followed by 35 cycles of 94 °C (20 s), 54 °C (20 s), 72 °C (30 s) with a final extension of 72 °C (5 min). Second round PCR cycling conditions consisted of one cycle of 94 °C for 3 min, followed by 25 cycles of 94 °C (20 s), 53 °C (20 s), 72 °C (30 s) with a final extension of 72 °C (5 min). Separation of amplified material and molecular weight markers (Bioline Hyperladder 100 bp) was performed by electrophoresis for 30 min at 100 mA on a 2.25% agarose gel pre-stained with ethidium bromide (Sigma-Aldrich, St. Louis, MO, USA) in 1× TAE (40 mM tris-acetate [pH 7.6], and 1 mM sodium EDTA). Gels were imaged under UV illumination using a Gel Doc (Biorad, Hercules, CA, USA).

For real time PCR, 2.5 µL of cDNA template was added to a final volume of 20 µL of commercially available fast master-mix (PerfeCTaqPCR FastMix, UNG, Low ROX, QuantaBio, Boston, MA, USA) containing MVE-specific primers (0.9 µM) and probe (0.2 µM) (Forward primer 5′-CAGGCCAGCCGGTTAGG, reverse primer 5′-GGTTCTGGGAGGCTTTCC, probe 5′[6FAM] CCAACCCCAGGAGGA[MGBNFQ]). Runs were performed on an Applied Biosystems (ABI) 7500 Fast Real-time PCR machine using standard cycling conditions of 95 °C for 20 s followed by 45 cycles of 95 °C (3 s), and 60 °C (30 s). The same procedure was followed for the BVDV internal control, (primers and probe sequences available on request).

A nuclease free water negative, and a virus positive control of low copy number were included for both conventional and Real-time PCR. 

MVEV detection was confirmed by sequencing the second round conventional pan-*Flavivirus* PCR amplified product. Reaction products were cleaned using the ExoSap-IT kit (Amersham Biosciences, Amersham, England) before sequencing using the ABI Prism Big Dye Terminator Cycle Sequencing Ready Reaction Kit. Primers used were the same as per the second round of amplification. Sanger sequencing was performed at Micromon (Monash University), using an ABI 3730 genetic analyser). Murray Valley encephalitis was confirmed by the use of GenBank Basic Local Alignment Tool (BLAST), (http://www.ncbi.nlm.nih.gov/Blast).

### 2.3. Virus Isolation

Virus isolation was performed by inoculating 500 µL of patient CSF onto a freshly confluent Vero cell monolayer in a T-25 (NUNC) tissue culture flask at 80% confluency: Cells were washed with PBS and left at 37 °C with inoculum for 30 min before the addition of 10 mL Eagle’s minimal essential medium containing 2% fetal bovine serum and incubation at 37 °C. Cells were monitored for cytopathic effect (CPE) and MVEV replication was confirmed by specific real-time PCR demonstrating increasing RNA copies.

### 2.4. Whole Genome Sequencing

A next-generation sequencing library was produced from 100 ng total RNA from the first passage of MVEV infected Vero cells using the Scriptseq V2 RNASeq library preparation kit (Illumina, San Diego, CA, USA) minus the rRNA depletion step. In brief, purified RNA was fragmented using heat and the RNA fragmentation solution to produce an average RNA fragment size of 300 bp. Reverse transcription of fragmented RNA with random hexamer oligonucleotide primers was performed to produce cDNA. The cDNA was tagged at both the 5′ and 3′ ends before second strand cDNA synthesis was performed. The di-tagged DNA molecules were then amplified by PCR for 15 cycles. The amplified DNA was then analyzed using the labchip GX platform to determine both quantity and size distribution of the library, before sequencing using an Illumina MiSeq V3 600 cycle PE kit according to the manufacturer’s instructions (Illumina, San Diego, CA, USA).

### 2.5. Bioinformatic Analysis

A consensus genome sequence for VIDRL_MVE (Genbank accession ID: MG452954) was established by mapping sequence reads in single-end mode to the 611W-WA-08 reference genome (Genbank accession: KM259934; length: 10,760 bp) and calling core genome single nucleotide polymorphism (SNP) differences using Snippy v3.2 (https://github.com/tseemann/snippy). The process was repeated against the same reference genome for five other publicly available MVEV genome sequences (MK6684, MVE-1-51, NG156, OR156, and V11-10) (Genbank accession: KF751869, AF161266, KF751870, KF751871, and JX123032 respectively) using the -ctgs flag in Snippy. The Snippy-core function was then used to prepare a multi-genome alignment file in fasta format from the individual mapping output folders for the six genomes. The resulting whole genome alignment file was the input for inferring the phylogenetic relationship among the MVEV genomes using FastTree v2.1.8 under a GTR model of nucleotide substitution. The tree was visualized with FigTree v1.4.3 (http://tree.bio.ed.ac.uk/software/figtree/). The sequence reads for VIDRL-MVEV have been deposited in GenBank Accession ID: MG452954.

## 3. Results

Positive results were obtained for the CSF sample in both the pan-*Flavivirus* conventional PCR, and the MVE-specific real-time PCR. The Cycle threshold (Ct) for the MVE-specific PCR was 41, consistent with a low virus titre. The internal control virus Ct was within normal limits indicating there was no inhibition of the PCR. 

Despite the likely low titre of virus, isolation of MVEV in Vero cells was achieved. CPE was observed on day 5 with morphology consistent with flaviviruses. Confirmation was performed by Real Time PCR which indicated a continuing decrease in Ct (from 21 to 17 over 5 days). This permitted extraction of sufficient RNA for whole genome sequencing.

A consensus genome sequence for VIDRL-MVE (Genbank Accession ID: MG452954) was prepared using approximately 10 million virus-specific sequencing reads mapped against the 10,760 bp 611W-WA-08 Genotype-1 reference genome. VIDRL-MVE differed from the reference genome by 772 Single Nucleotide Polymorphisms (SNPs) distributed throughout the genome, including the 5′ and 3′ UTRs (Figure S1A,B).

**Figure 1:**
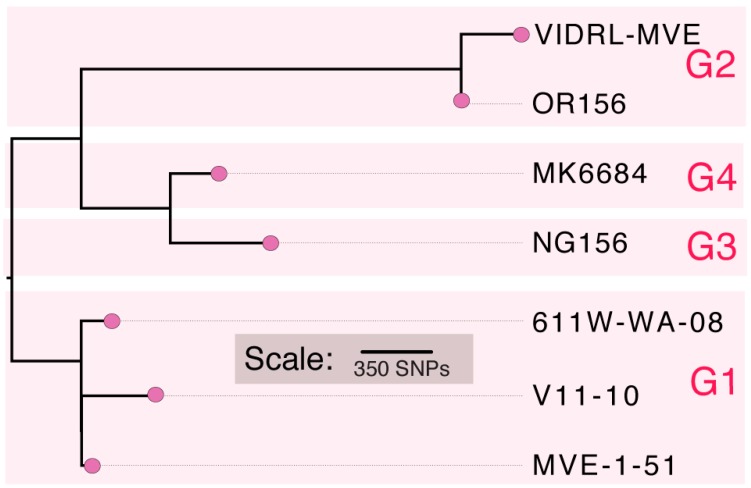
Maximum Likelihood tree (mid-point rooted) showing the phylogenetic relatedness of publicly available Murray Valley Encephalitis virus (MVEV) full genome sequences in comparison to VIDRL-MVEV. Isolate 611-WA-08 was used as the reference strain. Viral genotype (G1–G4) is also shown next to each taxon. OR156 is the G2 ancestral reference strain. All nodes had FastTree support values >0.95.

**Figure 2:**
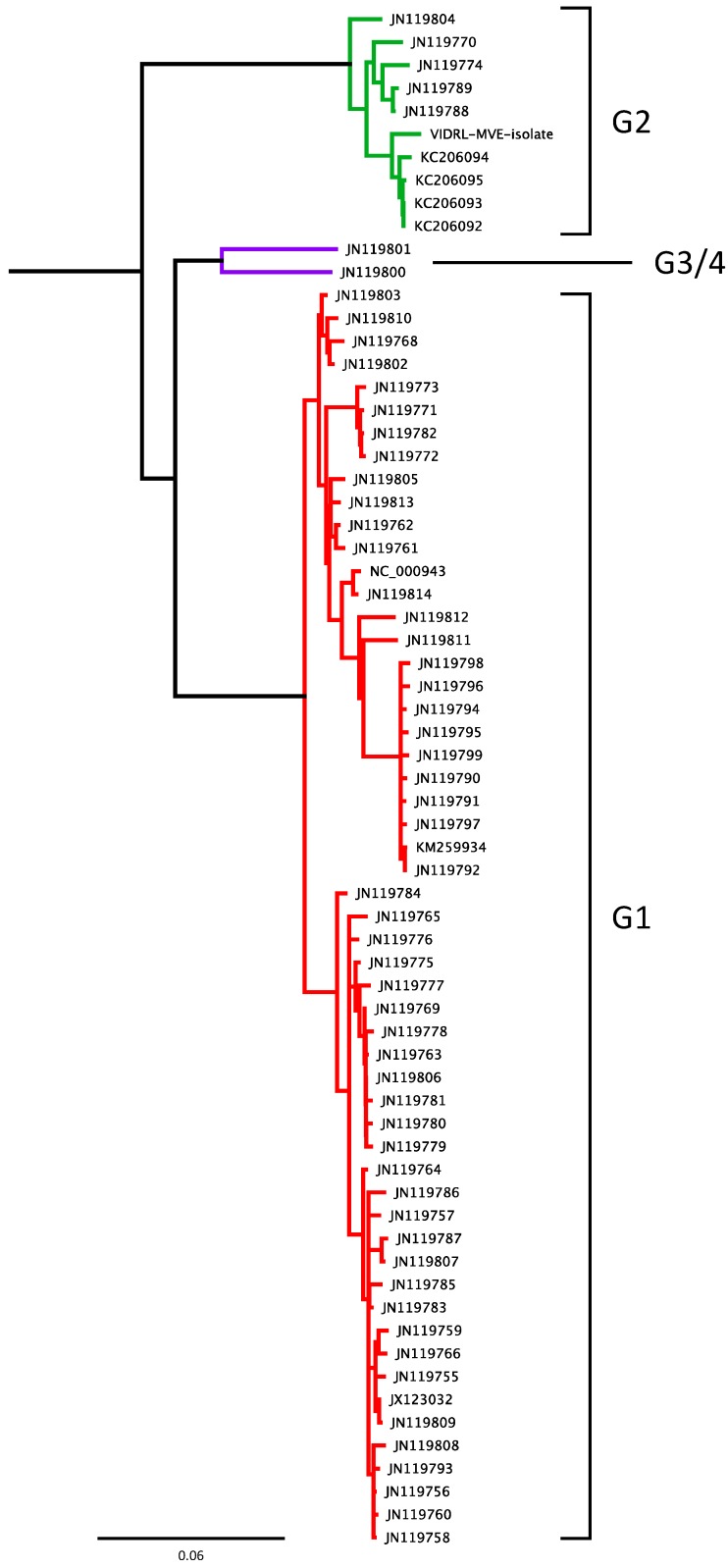
Neighbor joined tree of MVEV prM-E sequences confirm VIDRL-MVEV as Genotype 2. Viral genotype (G1–G4) is indicated. Scale bar indicates 0.06 nucleotide substitutions per site.

A phylogenetic analysis of the VIDRL-MVE genome in comparison to the five other published full genome sequences, representing isolates 611W-WA-08, MVE-1-51, MK6684, NG156 and OR156, was performed (Figure 1). This analysis, which used all 1730 variable nucleotides positions across the viral genome, showed that VIDRL-MVE was most closely related to MVE-OR156, a Genotype 2 virus isolated from mosquitoes in the Kimberley region of Australia in 1973. VIDRL-MVE was more distantly related to the Genotype 1, 3 and 4 viruses, having an estimated 1030 SNPS in comparison to the prototype MVEV isolate MVE-1-51. Therefore, approximately 10% of the VIDRL-MVE genome is divergent relative to the Genotype 1 virus MVE-1-51. Additionally, a phylogenetic tree was constructed from all available MVEV sequences corresponding to the prM-E gene(s) (~2000 bp region) to further expand Figure 1 and confirm our isolate as genotype 2 (Figure 2).

A more detailed comparison of MVE-OR156 and VIDRL-MVE identified 288 variable nucleotide positions between VIDRL-MVE and MVE-OR156 resulting in 31 predicted amino acid changes distributed throughout the viral genome (Table 1). The biological significance of these changes remains unknown.

## 4. Discussion

Here we present the first known isolation and subsequent full genome sequencing of MVEV from CSF. 

Historically MVEV diagnosis by isolation and/or PCR has been difficult due to factors such as low viral burden, limited availability of appropriate samples, and infrequency of cases. The last report of multiple isolations from human specimens was from the 1974 outbreak where isolation was attempted on a number of samples, (including 11 throat swabs and 14 CSF) from 24 patients, 22 of which were admitted to our laboratory, then located at Fairfield hospital [7]. In that report, MVEV was only successfully isolated from post-mortem brain tissue, (specifically 6 samples from 3 patients). The systems utilized to cultivate the virus were 11–12 days embryonated eggs, cell lines (Monkey Embryonic Kidney, (MEK) and Vero) and newborn mice. While eggs were the most sensitive method, MVEV was also successfully cultured in MEK cells from two samples (one of which also grew in Vero). 

More recently, isolation of MVEV from post-mortem brain tissue of a deceased patient from the 2011 outbreak has also been described [8].

Isolation of the MVEV from cell culture of the clinical specimens has not subsequently been reported, perhaps reflecting that techniques of virus isolation have given way to PCR due to its increased speed, sensitivity and specificity, and reduced demand for specific expertise compared to tissue culture. In contrast, this case highlights that cell culture may be more achievable than has been assumed, and supports its use in MVEV genotyping and investigation. The clinical significance of MVEV isolation from a CSF sample with low levels of virus is two-fold. Firstly, it contradicts the perceived superiority (sensitivity) of PCR. We were able to isolate MVEV from CSF yielding a low level Real-Time PCR detection (Ct 41) by inoculating with a large volume. Despite requiring a minimum sample volume of 140–200 µL for nucleic acid extraction, PCR only allows for an input that is equivalent to approximately 4–10 µL of that original sample. By comparison, our inoculum volume was 500 µL, allowing for a higher input of virus particles, thereby potentially increasing the relative sensitivity of the technique when close to the limit of detection.

PCR detection and isolation of MVEV from CSF emphasizes its usefulness as a sample for primary diagnosis, with downstream applications for whole genome sequencing. CSF is far preferable to brain tissue, (often acquired post-mortem) and more sensitive than plasma, which must be obtained before the appearance of antibodies clear the viremia [3], consequently, when plasma is no longer useful, CSF may still present as a viable option. 

Furthermore, in instances where original samples yield very low PCR-positive results, isolation provides a means for generating enough RNA for whole genome sequencing. This in turn provides valuable information for use in epidemiology as well as virological/phylogenetic investigations. Currently four genotypes of MVEV are recognized, however full genome sequencing has only been performed on five isolates, four of which are from at least 40 years ago [1]. Thus, information on today’s portrait of circulating MVEV and how this pertains to clinical outcomes is limited. 

A recent study by Williams et al., 2015 described the sequencing of smaller gene fragments including pre-membrane and envelope genes from 66 strains of MVEV from 1951 to 2011 [5]. The paper confirmed that Genotype 1 is the predominant genotype in Australia and revealed that more recent strains of MVEV belong to 2 distinct sub-lineages of Genotype 1: G1A and G1B, of which G1B is more geographically diverse and most widely transmitted, leading to suggestions that it may have a replicative advantage.

In light of this data, as well as previous findings by Williams et al., 2015 [5] where all Genotype 2 strains originated from mosquito isolates in the north east Kimberly region of Western Australia, our identification of Genotype 2 in recent, human derived MVEV from the Northern Territory is somewhat surprising. 

The current profile of MVEV Genotype 2 describes a less dominant species. Suggested to be initially introduced from Papua New Guinea, Genotype 2 has either become more established over time, or been repeatedly re-introduced. The geographically confined nature of Genotype 2 has led to suggestions that it inhabits an ecological niche, [5] while mouse models demonstrate it to be phenotypically less virulent compared to the dominant MVEV Genotype 1 [13]. Additionally, it has been demonstrated that time, rather than geographical location, is the main predictor for all circulating MVEV genotypes, (i.e., all strains over a geological range at a single time point are related [14]).

Thus, while the low numbers of clinical MVEV cases makes it difficult to draw definitive conclusions regarding the significance of this case the following questions arise: Does the appearance of MVEV Genotype 2 causing human disease reflect a single ‘rare’ incidence or importation; or is Genotype 2 becoming a more dominant representative of MVEV currently circulating? Considering the high proportion of MVEV infections that remain asymptomatic it is realistic to suggest that this single case could represent just one out of hundreds of undetected infections. Furthermore, the detection in humans may mean that this current Genotype 2 is more virulent than previously believed. Whether this virulence is due to genetic evolution of local MVEV or the result of an import is yet to be determined.

## Figures and Tables

**Table 1:** Comparison of VIDRL-MVE to the G2 reference strain MVE-OR156 indicating the 31 predicted non-synonymous amino acid changes.

Amino Acid Change	Protein	Non-Structural Protein Function
P11S	core protein	
A10T	signal peptide	
S7G	envelope protein	
V68I	envelope protein	
L311F	envelope protein	
R33K	NS1 protein	Early replication [12]
V188I	NS1 protein	
D240E	NS1 protein	
F1L	NS2A protein	replicase component, genome encapsidation [12]
S93T	NS2A protein	
I197V	NS2A protein	
D27E	NS2B protein	NS3 cofactor. Protease cofactor involved in infectious virus production [12]
N59D	NS2B protein	
A67T	NS2B protein	
S106G	NS2B protein	
I110T	NS2B protein	
P131S	NS3 protein	Genome encapsidation, capsid cleavage, protease, Ntpase, helicase [12]
V241I	NS3 protein	
G321S	NS3 protein	
I54V	NS4A protein	NS3 cofactor [12]
L69F	NS4A protein	
D98E	NS4A protein	
L21F	NS4B protein	RNA accumulation [12]
L32F	NS4B protein	
T69N	NS4B protein	
N76S	NS4B protein	
A115V	NS4B protein	
R101K	NS5 protein	RNA polymerase, methyltransferase [12]
T116M	NS5 protein	
I374T	NS5 protein	
K403E	NS5 protein	

## Supplementary Materials

The following are available online at http://www.mdpi.com/1999-4915/10/6/319/s1, Figure S1: Genomic Analysis of VIDRL-MVE.
