# Supplementary material for: The First Isolation and Whole Genome Sequencing of Murray Valley Encephalitis Virus from Cerebrospinal Fluid of a Patient with Encephalitis
Source: Viruses. 2018 Jun 11;10(6):319. doi: 10.3390/v10060319 (PMC6024754; doi:10.3390/v10060319)
Supplement: Supplementary file 1 [file viruses-10-00319-s001.pdf]

Fig. S1: Genomic analysis of VIDRL-MVEV.

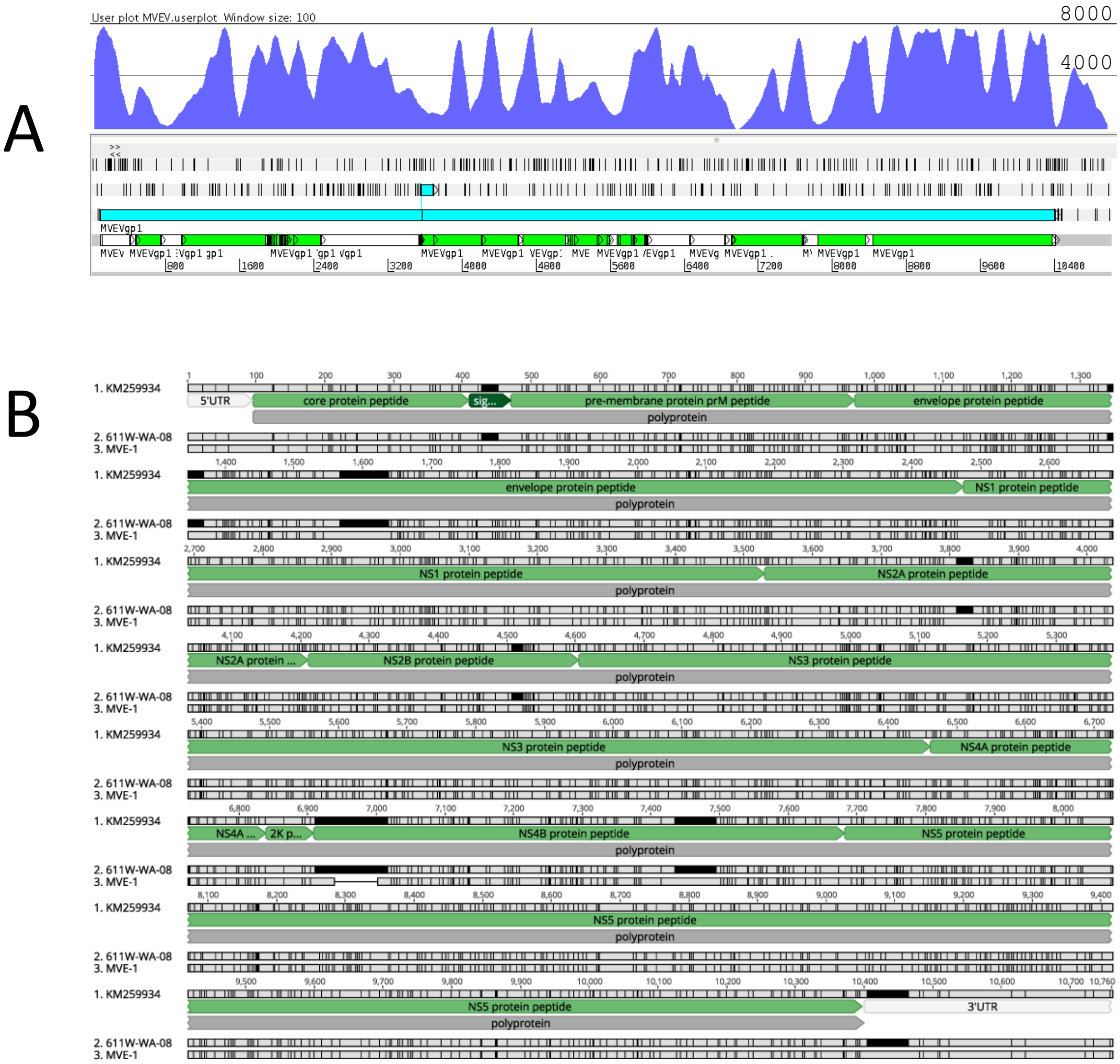

Fig. S1: Genomic analysis of VIDRL-MVEV. (A). Distribution of reads mapped to the 611W-WA-08 reference sequence. Upper element shows read coverage plot. (B) Distribution of SNPs in VIDRL-MVEV genome compared to the reference.
